# Identification of novel autophagy-related lncRNAs associated with a poor prognosis of colon adenocarcinoma through bioinformatics analysis

**DOI:** 10.1038/s41598-021-87540-0

**Published:** 2021-04-13

**Authors:** Dejun Wu, Zhenhua Yin, Yisheng Ji, Lin Li, Yunxin Li, Fanqiang Meng, Xiaohan Ren, Ming Xu

**Affiliations:** 1 Department of General Surgery, Shanghai Pudong Hospital, Fudan University Pudong Medical Center, 2800 Gongwei Road, Shanghai, 201399 China; 2 Department of Digestive, Shanghai Pudong Hospital, Fudan University Pudong Medical Center, 2800 Gongwei Road, Shanghai, 201399 China; 3 The State Key Lab of Reproductive, Department of Urology, The First Affiliated Hospital of Nanjing Medical University, Nanjing, 210029 China; 4 First Clinical Medical College, Nanjing Medical University, Nanjing, 210029 China; 5 Xiangya Medical College of Central South University, Changsha, 410000 Hunan China

**Keywords:** Cancer, Computational biology and bioinformatics, Genetics

## Abstract

LncRNAs play a pivotal role in tumorigenesis and development. However, the potential involvement of lncRNAs in colon adenocarcinoma (COAD) needs to be further explored. All the data used in this study were obtained from The Cancer Genome Atlas database, and all analyses were conducted using R software. Basing on the seven prognosis-related lncRNAs finally selected, we developed a prognosis-predicting model with powerful effectiveness (training cohort, 1 year: AUC = 0.70, 95% Cl = 0.57–0.78; 3 years: AUC = 0.71, 95% Cl = 0.6–0.8; 5 years: AUC = 0.76, 95% Cl = 0.66–0.87; validation cohort, 1 year: AUC = 0.70, 95% Cl = 0.58–0.8; 3 years: AUC = 0.73, 95% Cl = 0.63–0.82; 5 years: AUC = 0.68, 95% Cl = 0.5–0.85). The VEGF and Notch pathway were analyzed through GSEA analysis, and low immune and stromal scores were found in high-risk patients (immune score, cor =  − 0.15, *P* < 0.001; stromal score, cor =  − 0.18, *P* < 0.001) , which may partially explain the poor prognosis of patients in the high-risk group. We screened lncRNAs that are significantly associated with the survival of patients with COAD and possibly participate in autophagy regulation. This study may provide direction for future research.

## Introduction

Colon adenocarcinoma (COAD) is not only the third most common tumor but also the fourth leading cause of tumor-related death worldwide; over one million cases of the disease are reported, resulting in approximately 700,000 deaths^1,2^. COAD has been suggested to be associated with obesity-induced changes in tissue microenvironments, imbalance in the microbiome, and inflammation^3–5^. Although the main treatments for COAD, including surgical excision and chemoradiotherapy, could alleviate COAD at the early stages, patients with advanced colon cancer still have poor prognoses due to tumor metastasis. Therefore, research identifying critical regulators in carcinogenesis is essential and may provide new tumor markers and drug targets and eventually improve the therapeutic efficiency of COAD treatment.

Autophagy is a catabolic process in which proteins and whole organelles in cells degenerate as a result of increase in lysosomes and can be activated in physiological and pathological conditions. On the one hand, autophagy can provide necessary circulating metabolic substrates for survival in response to stress under normal circumstances. On the other hand, autophagy occurs in some pathological processes for the maintenance of cellular homeostasis, such as aging, cancer pathogenic inflammation, and neurodegenerative diseases^6^. Moderate and active autophagy can remove tumor cells and thereby regulate homeostasis, whereas impaired autophagy may delay the elimination of apoptotic cells from the body, triggering the development of cancer. Xiao et al. found that the progressive up-regulation of autophagy-related protein RACK1 in the carcinogenic process of human colonic epithelium might be involved in the oncogenesis of COAD^7^. Kim et al. revealed that ECZ-induced autophagy in mouse COAD CT-26 cells can be used in cancer chemoprevention or cancer chemotherapy^8^.

LncRNAs, defined as non-protein-coding RNA transcripts longer than 200 nucleotides (nt), play an essential role in diverse cellular processes, including RNA decay, genetic regulation of gene expression, RNA splicing, microRNA regulation, and protein folding^4^. As such, lncRNAs and their regulatory effects are intensively investigated. The role of lncRNAs in regulating the proliferation, apoptosis, migration, invasion, and chemoresistance of COAD cells has been explored^8,9^. For example, Liang et al. showed that the expression of lncRNA B3GALT5-AS1 decreases in COAD and liver metastasis tissues^10^. Additionally, another experiment indicated that the lncRNA PVT1 can promote the migration, invasion, and multiplication of COAD by sponging miR-26b^11^.

In recent years, high-throughput platforms for gene expression have been developing rapidly and widely used in many fields, such as molecular classification, prognosis prediction, and targeted drug discovery^12^. The broad discipline of bioinformatics can be used in capturing, storing, analyzing, and interpreting biological data with specific algorithms and software. Here, we identified seven autophagy-related lncRNAs that may significantly affect the prognosis of tumor patients and established a prognosis prediction model based on these lncRNAs. A Nomogram plot was constructed for a better application in clinical. Overall, our result delineated the role of autophagy-related lncRNAs in COAD, which might be novel targets for cancer therapy.

## Materials and methods

### Data acquisition and processing

Independent RNA-seq data, including the gene profile information of mRNA and lncRNAs, were obtained from the The Cancer Genome Atlas (TCGA) database. Corresponding clinical and prognosis information was also acquired. The file “Homo_sapiens.GRCh38.99.chr.gtf,” which is available in the Ensembl website was used for gene annotation^13^. Data processing involved background correction, data normalization, batch effect removal, and combining of normal and tumor group data, which were carried out using R software. All the data were obtained from an open-access database, and therefore no approval was needed from the Medical Ethics Committee.

### Autophagy-related genes and lncRNA screening

The autophagy gene list was obtained from the Human Autophagy Database (HADb, http://autophagy.lu/clustering/index.html), which is the first human autophagy-dedicated database and a public repository containing information about the human genes currently known to be involved in autophagy. Then, we extracted the autophagy gene data from the TCGA-COAD mRNA expression profile using the “limma” package in R software and identified lncRNA related to autophagy genes. Only lncRNAs with correlation coefficient |R^2^|> 0.3 and *P* < 0.001 were considered autophagy-related lncRNAs.

### Construction and validation of the prognosis prediction model

We first randomly distrubuted the samples from the TCGA database into training and test cohorts (1:1). After combining the lncRNA expression profile with the prognosis data, we performed univariate cox analysis, LASSO regression, and multivariate cox analysis in sequential order to screen prognosis-related lncRNAs. Then, a prognosis prediction model was established with the formula Risk scores = ΣCoef * *exp*(*genes*). The calculation was performed with the “survival” package in R software and validated in the test cohort. Patients with risk scores above the median were included in the high-risk group, and others were included in the low-risk group. Univariate and multivariate analyses were used in estimating the independence of the prognosis model. Meanwhile, ROC curves were used in detecting the effectiveness of our prognosis model. The prognostic roles of each prognosis-related lncRNAs were tested using Kaplan–Meier curves.

### Co-expression network and enrichment analysis

Based on the seven prognosis-related lncRNAs identified, the co-expression network linking these autophagy-related lncRNAs and autophagy-related genes was established. Cytoscape (version 3.4; The Cytoscape Consortium (San Diego, CA, USA) was then used in visualizing the PPI networks^14^. The “clusterProfiler” package in R software was used in GO and KEGG enrichment analyses (www.kegg.jp/kegg/kegg1.html)^15^. A P value of < 0.05 was regarded as statistically significant, and the top ten results of enrichment analyses were selected for visualization.

### Nomogram and calibration

We included the clinical features of age, gender, stage, and TNM classification in our analysis and considered the practical utility of our model in clinical processes. A nomogram plot was constructed using the “rms” package in R software for the prediction of 1-, 3-, and 5-year survival rates of patients with COAD. Calibration plots were used in assessing the prognostic accuracy of the nomogram. Specifically, nomogram-predicted and actual probabilites of the patients were compared.

### Gene set enrichment analysis and gene set variation analysis

After the samples were divided into high- and low-risk score groups, GSEA was conducted for the link the genes to feasible pathways^16^. Gene set permutations were performed 1000 times for each analysis. The enriched pathways were selected under the following screening conditions: FDR < 0.25 and NOM *P* value < 0.05. Gene set variation analysis (GSVA) was performed using the GSVA package R software with the Hallmark dataset.

### Clinical correlation and tumor microenvironment analysis

Wilcox test was used in investigating the clinical relevance of the seven lncRNAs, risk scores, and clinical features. The “Estimate” package and ssGSEA algorithm were used in the calculation of stromal and immune scores in tumor microenvironment.

### Cell lines and qPCR

Normal human colon mucosal epithelial cell line (CCD-18Co) and the human COAD cell lines (SW480, LS174T, HCT116, DLD-1 and HT29) were purchased from iCell (Shanghai, China). Total RNA was isolated using Trizol (Invitrogen). PrimeScript RT Master Mix (Takara, JPN) was used for cDNA synthesis. The primers used were: LINC01063, forward: 5′-TATCAAGCGGTGGCAGTTCAGC-3′; LINC01063, reverse: 5′-GCCAATCACCTTCCAGGCTCA-3′; MIR210HG, forward: 5′-AGCTGGGCAGACAGGAGTGAAGT-3′; MIR210HG, reverse: 5′-AGGCAACTCGGCTTGGTTATTTC-3′; AC027307.2, forward: 5′-AAACTGCTGGGATTACAGGTATGAGC-3′; AC027307.2, reverse: 5′-CCAGAAGGGCAAAGATAGATAGAAGACA-3′; AC073611.1, forward: 5′-CACCACGATGTCACAGGAAGC-3′; AC073611.1, reverse: 5′-GGGAGGATGAGGCAGGAGA-3′; AC156455.1, forward: 5′-TCTGGGCTCCCTCCGTGAT-3′; AC156455.1, reverse: 5′-ACCCTGTCCAAGTCGCTTCC-3′; PCAT6, forward: 5′-CACCGGCTTTCCCTCGTCCTCT-3′; PCAT6, reverse: 5′-CGCAAGCGTTTGTGGGTTTCA-3′; AL161729.4, forward: 5′-TGTATTCCTACAACACCCAGAC-3′; AL161729.4, reverse: 5′-TGTGCCTCCTAGCAAACG-3′; GAPDH, forward: 5′-ACCACAGTCCATGCCATCAC-3′; GAPDH, reverse: 5′-TCCACCACCCTG TTGCTGTA-3′;

## Result

### Screening for autophagy-genes and -lncRNAs

**Figure 1:**
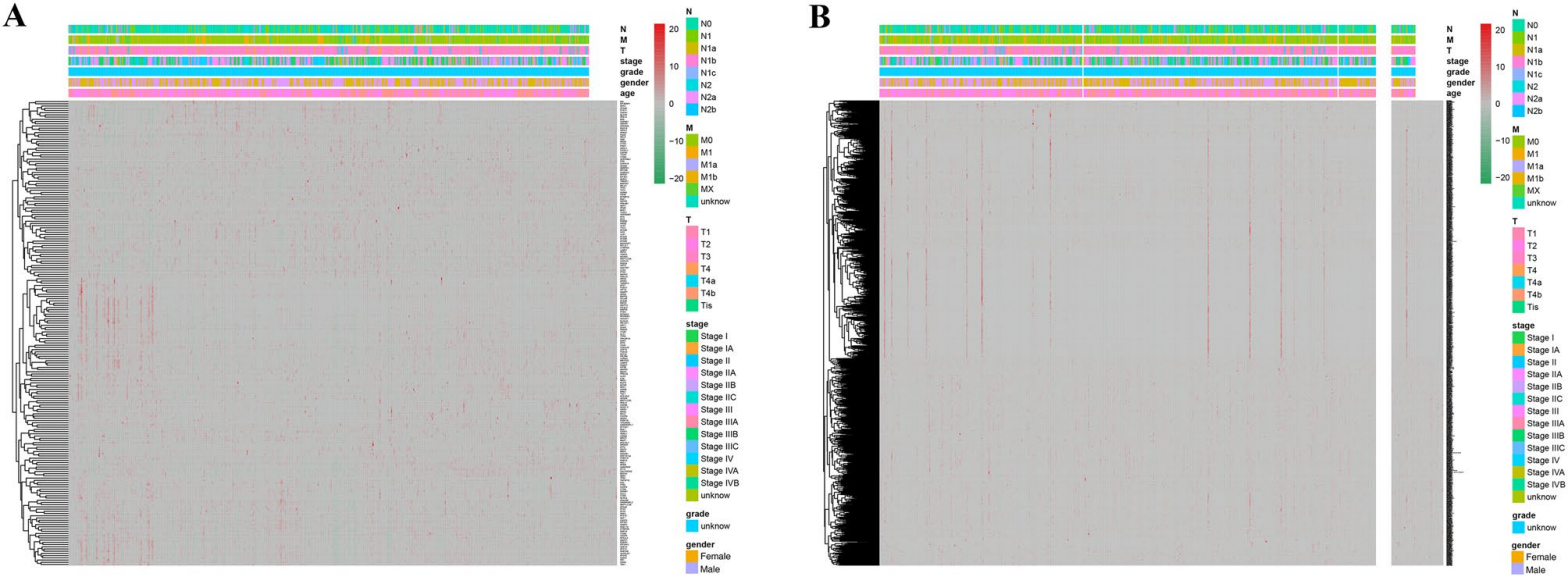
The heatmap of autophagy-related lncRNAs and genes in TCGA cohort.

A total of 231 autophagy-related genes were identified from the website of HADb (Table S1 and Fig. 1A), then 1285 autophagy-related lncRNAs were screened at a set threshold of correlation coefficient |R^2^| of > 0.3 and *P* value of < 0.001 (Fig. 1B).

### Construction of the prognosis model and co-expression network

**Figure 2:**
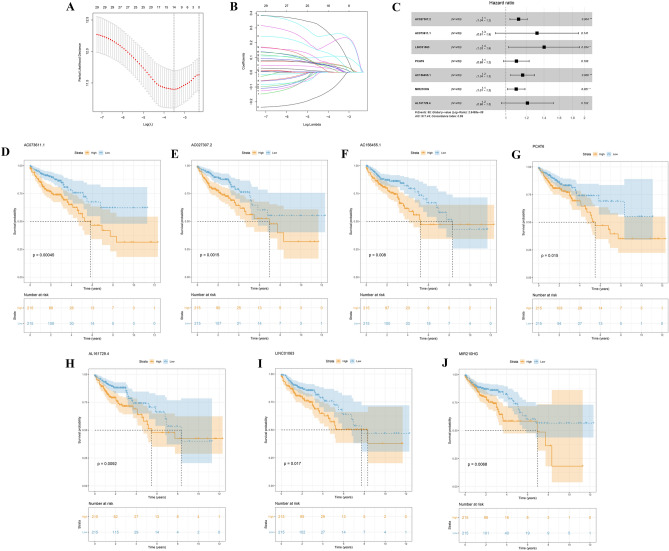
Identification of the prognosis-related lncRNAs. (**A&B)** LASSO coefficient profiles; (**C**) Multivariate cox analysis of seven model lncRNAs; (**D**) The Kaplan–Meier curves of AC073611.1; (**E)** The Kaplan–Meier curves of AC027307.2; (**F**) The Kaplan–Meier curves of AC156455.1; (**G**) The Kaplan–Meier curves of PCAT6; (**H**) The Kaplan–Meier curves of AL161729.4; (**I**) The Kaplan–Meier curves of LINC01063; (**J**) The Kaplan–Meier curves of MIR210HG.

**Figure 3:**
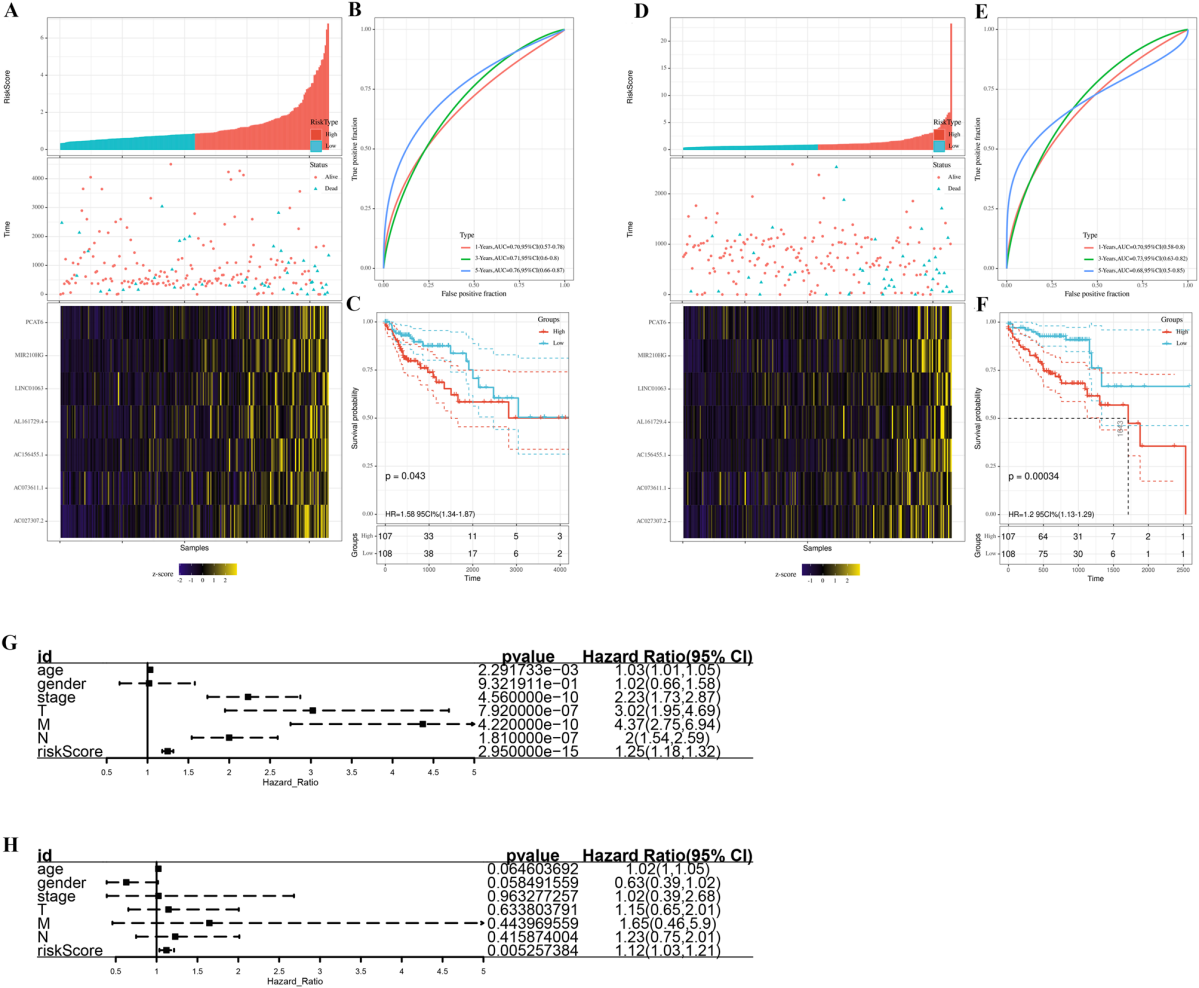
Construction and validation of the prognosis model. (**A**) The risk scores of patients in TCGA training cohort; (**B**) ROC curve of patients in TCGA training cohort; (**C**) Kaplan–Meier curve of patients in TCGA training cohort; (**D**) The risk scores of patients in TCGA validation cohort; (**E**) ROC curve of patients in TCGA validation cohort; (**F**) Kaplan–Meier curve of patients in TCGA validation cohort; (**G**) Univariate analysis of model and clinical features; (**H**) Multivariate analysis of model and clinical features. TCGA, The Cancer Genome Atlas.

According to the survival data in TCGA, we performed univariate cox analysis, LASSO regression, and multivariate cox analysis on the 1285 autophagy-related lncRNAs. Seven lncRNAs were considered related to prognosis (*P* < 0.05; Fig. 2A–C), namely, AC027307.2, AC073611.1, LINC01063, PCAT6, AC156455.1, MIR210HG, and AL161729.4, and were used in establishing a prognosis model. The result of Kaplan–Meier curves indicated that all the seven lncRNAs were associated with poor prognosis (Fig. 2D–J). The patients were included in high- or low-risk group according to their risk scores, which were computed using the formula AC027307.2* 0.11 + AC073611.1* 0.27 + LINC01063* 0.33 + PCAT6* 0.09 + AC156455.1* 0.15 + MIR210HG* 0.09 + AL161729.4* 0.19 (Table 1). Figure 3A demonstrates the expression of the seven lncRNAs in the high- or low-risk group (TCGA training cohort). The area under the ROC curve (AUC) values of the risk scores were all > 0.7, showing the effectiveness of our prognosis model (Fig. 3B; 1 year: AUC = 0.70, 95% Cl = 0.57–0.78; 3 years: AUC = 0.71, 95% Cl = 0.6–0.8; 5 year: AUC = 0.76, 95% Cl = 0.66–0.87). Compared with the low-risk group, the Kaplan–Meier curves exhibited poor prognosis in the high-risk group (Fig. 3C; *P* = 0.043). In the TCGA validation cohort (Fig. 3D), our model demonstrated satisficatory performance in the ROC curve (Fig. 3E; 1 year: AUC = 0.70, 95% Cl = 0.58–0.8; 3 years: AUC = 0.73, 95% Cl = 0.63–0.82; 5 years: AUC = 0.68, 95% Cl = 0.5–0.85) and Kaplan–Meier curves (Fig. 3F; *P* = 0.00034). The basic clinical features of COAD patients in training and validation cohort are shown in Table 2. Moreover, the P value in a single-factor (Fig. 3G; HR = 1.173, 95% Cl: 1.125–1.223) and multiple-factor (Fig. 3H; HR = 1.149, 95% Cl: 1.098–1.202) analyses were less than 0.01, indicating that the prognosis model is independent of other clinical factors (TNM stage, clinical stafe, age, and gender) and significantly associated with the prognosis of patients.

**Table 1:** The 7 lncRNAs for construction of prognosis model.

lncRNAs	Coef^a^	HR^b^
AC027307.2	0.11	1.11
AC073611.1	0.27	1.31
LINC01063	0.33	1.40
PCAT6	0.09	1.09
AC156455.1	0.15	1.16
MIR210HG	0.09	1.09
AL161729.4	0.19	1.21

^a^Coefficients.

^b^Hazard ratios.

**Table 2:** The basic characteristics of the clinical variables in COAD patients.

Clinical features	Total	Training cohort	Validation cohort
Sample size	430	215	215
**Age**			
< = 65	173	83	90
> 65	257	132	125
**Gender**			
Female	206	107	99
Male	224	108	116
**Stage**			
Stage I	73	39	34
Stage II	173	84	89
Stage III	123	62	61
Stage III	61	30	31
**T classification**			
T1	8	2	6
T2	73	42	31
T3	295	148	147
T4	54	23	31
**M classification**			
M0	326	169	157
M1	61	30	31
Mx	43	16	27
**N classification**			
N0	254	125	129
N1	100	46	54
N2	76	44	32

### Co-expression network and enrichment analysis

**Figure 4:**
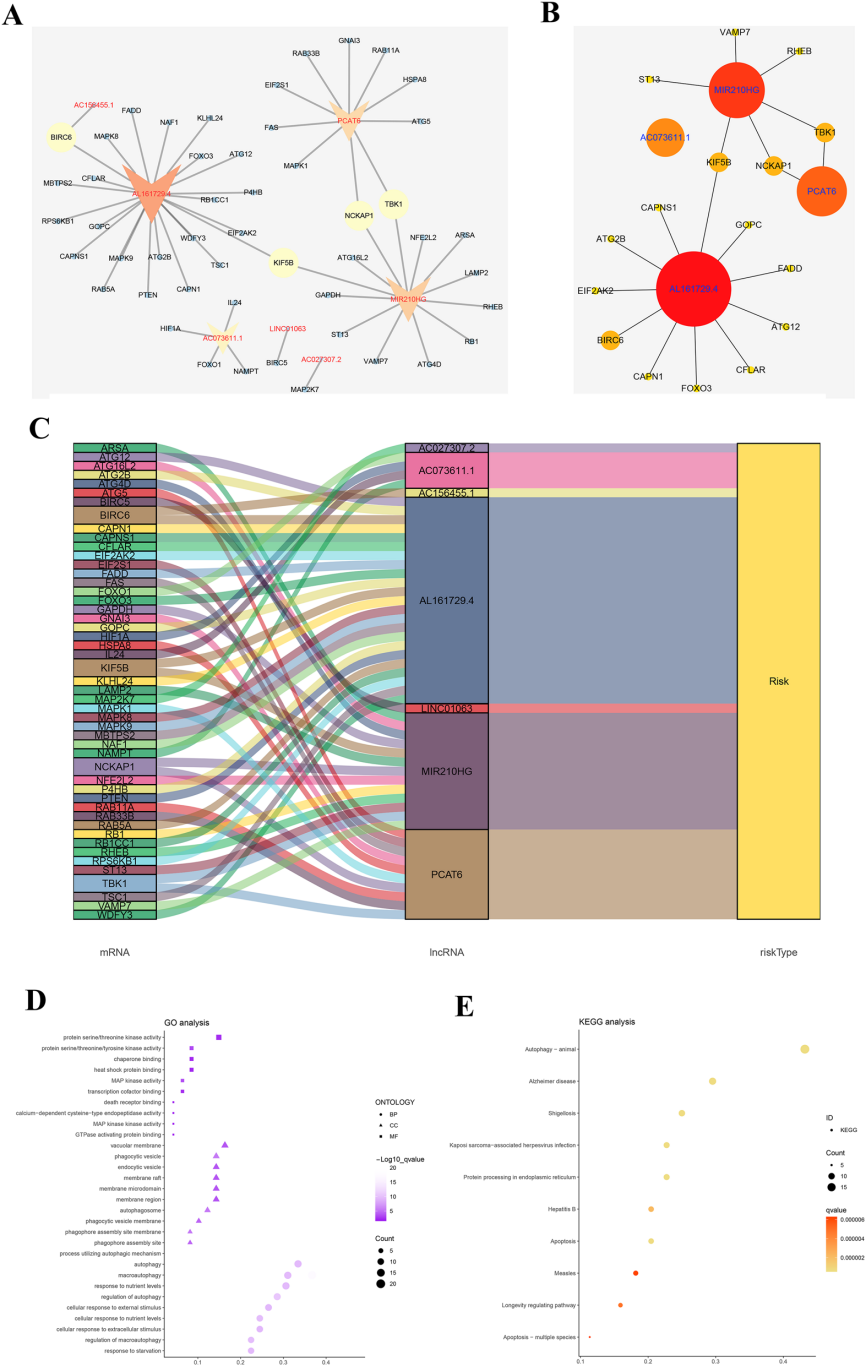
Co-expression network and enrichment analysis. (**A**) Edges and nodes in co-expression network; (**B**) Top 20 nodes in co-expression network; (**C**) Sankey diagram of lncRNAs and linked genes; (**D**) GO analysis of the genes in co-expression network**; (E**) KEGG analysis of the genes in the co-expression network. GO: Gene Ontology; KEGG: Kyoto Encyclopedia of Genes and Genomes.

A total of 47 autophagy-related genes were found to be associated with the seven prognosis-related lncRNAs. Furthermore, the co-expression network based on the relationships of these genes with the lncRNAs were established with 54 nodes and 51 edges (Fig. 4A and Figure S2). The top 20 significant nodes were selected according to the MCC value in the cytohubba analysis, and the lncRNA AL161729.4 was the most important node (Fig. 4B). The Sankey diagram intuitively showed the association of each node with patients' prognosis (Fig. 4C). GO and KEGG analyses were conducted for the functional enrichment of the nodes in this co-expression network (Fig. 4D–E). The result revealed that for biological processes, the nodes were mainly enriched in “autophagy,” “macro autophagy,” “response to nutrient levels,” and “regulation of autophagy.” Changes in cellular components were markedly enriched in “vacuolar membrane,” “phagocytic vesicle,” “endocytic vesicle,” and “membrane raft.” Changes in the DEG molecular function were primarily enriched in “protein serine/threonine kinase activity,” “protein serine/threonine/tyrosine kinase activity,” “chaperone binding,” and “heat shock protein binding.” KEGG analysis results showed that DEGs were strikingly enriched in “Autophagy-animal,” “Alzheimer’s disease,” “Shigellosis,” and “Kaposi sarcoma-associated herpes virus infection.”

### Nomogram and calibration

**Figure 5:**
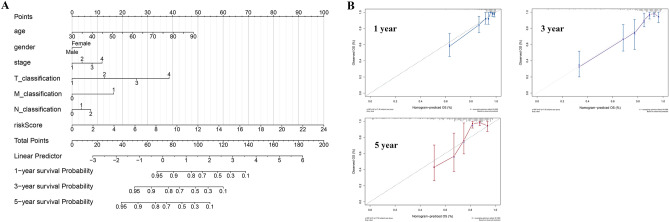
Construction of a nomogram based on risk score and clinical information. (**A**) The nomogram plot; (**B**) The calibrations of 1, 3, 5 years.

Multivariable cox proportional hazards analysis was performed for the construction of a nomogram based on clinical factors and risk scores (seven lncRNAs OS prediction model). The primary clinical factors included gender, age, stage, and TNM classification (Fig. 5A). The nomogram showed great effectiveness and stability when assessed with calibrations (Fig. 5B; 1 year, gray: ideal; 3 year, gray: ideal; 5 year, gray: ideal).

### GSEA and GSVA analysis

**Figure 6:**
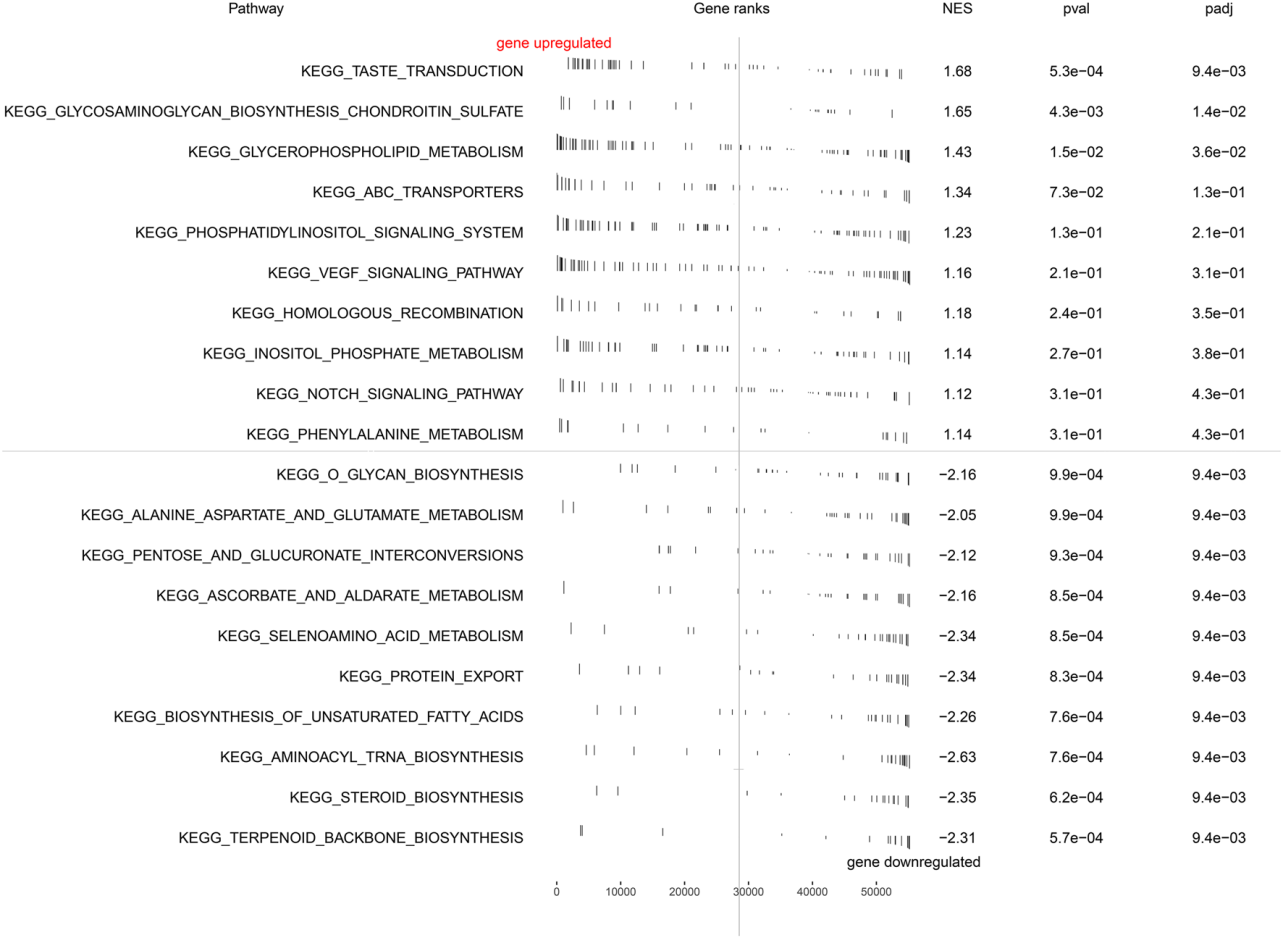
GSEA enrichment analysis.

The biological functions of the high- and low-risk groups were further explored through GSEA analysis. As is shown in Fig. 6, the VEGF and Notch signaling pathways were enriched in the high-risk phenotype. In the low-expression phenotype, the top five enriched gene sets were glycan biosynthesis, alanine aspartate and glutamate metabolism, pentose and glucuronate interconversions, ascorbate and aldarate metabolism, and metabolism of seven amino acids. The underlying signaling pathway of seven lncRNAs was analyzed through GSVA analysis (Figure S1).

### Clinical correlation and tumor microenvironment analysis

**Figure 7:**
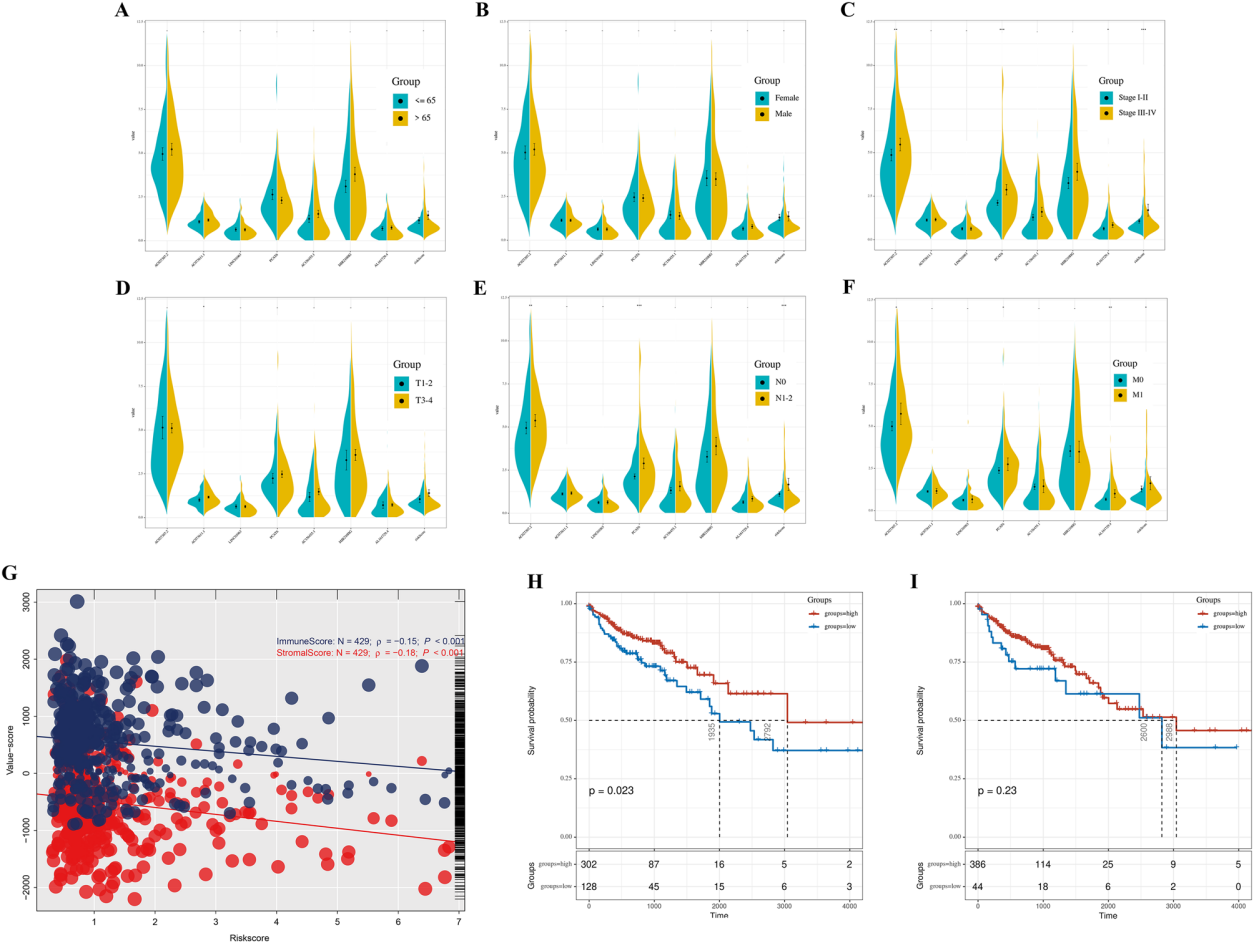
Clinical correlation and tumor microenvironment analysis. (**A**) The correlation of age with model lncRNAs and risk score; (**B**) The correlation of gender with model lncRNAs and risk score; (**C**) The correlation of clinical-stage with model lncRNAs and risk score; (**D**) The correlation of T classification with model lncRNAs and risk score; (**E**) The correlation of N classification with model lncRNAs and risk score; (**F**) The correlation of M classification with model lncRNAs and risk score; (**G**) The negative correlation of risk score with immune and stromal score. (**H**) Kaplan–Meier curve of the immune score in TCGA patients; (**I**) Kaplan–Meier curve of the stromal score in TCGA patient.

The associations of the expression levels of the seven lncRNAs with clinicopathological parameters were investigated (Fig. 7A–F). The result showed that high values of AC027307.2, PCAT6, AL161729.4, and risk scores were correlated with a worse clinical stage (stage III/IV; Fig. 7C); the high value of AC073611.1 was associated with poor T classification (T3/4; Fig. 7D); the high value of AC027307.2, PCAT6, and risk scores might lead to poor N classification (N1/2; Fig. 7E); the high values of AC027307.2, PCAT6, AL161729.4, and risk scores were related to poor M classification (M1; Fig. 7F). The risk score was found inversely proportional to the stromal and immune score of the tumor microenvironment (immune score, cor =  − 0.15, P < 0.001; stromal score, cor =  − 0.18, *P* < 0.001; Fig. 7G). Interestingly, decreases in stromal and immune scores were associated with poor prognosis in COAD patients (Fig. 7H–I).

### The mRNA expression of seven lncRNAs in COAD cell lines

**Figure 8:**
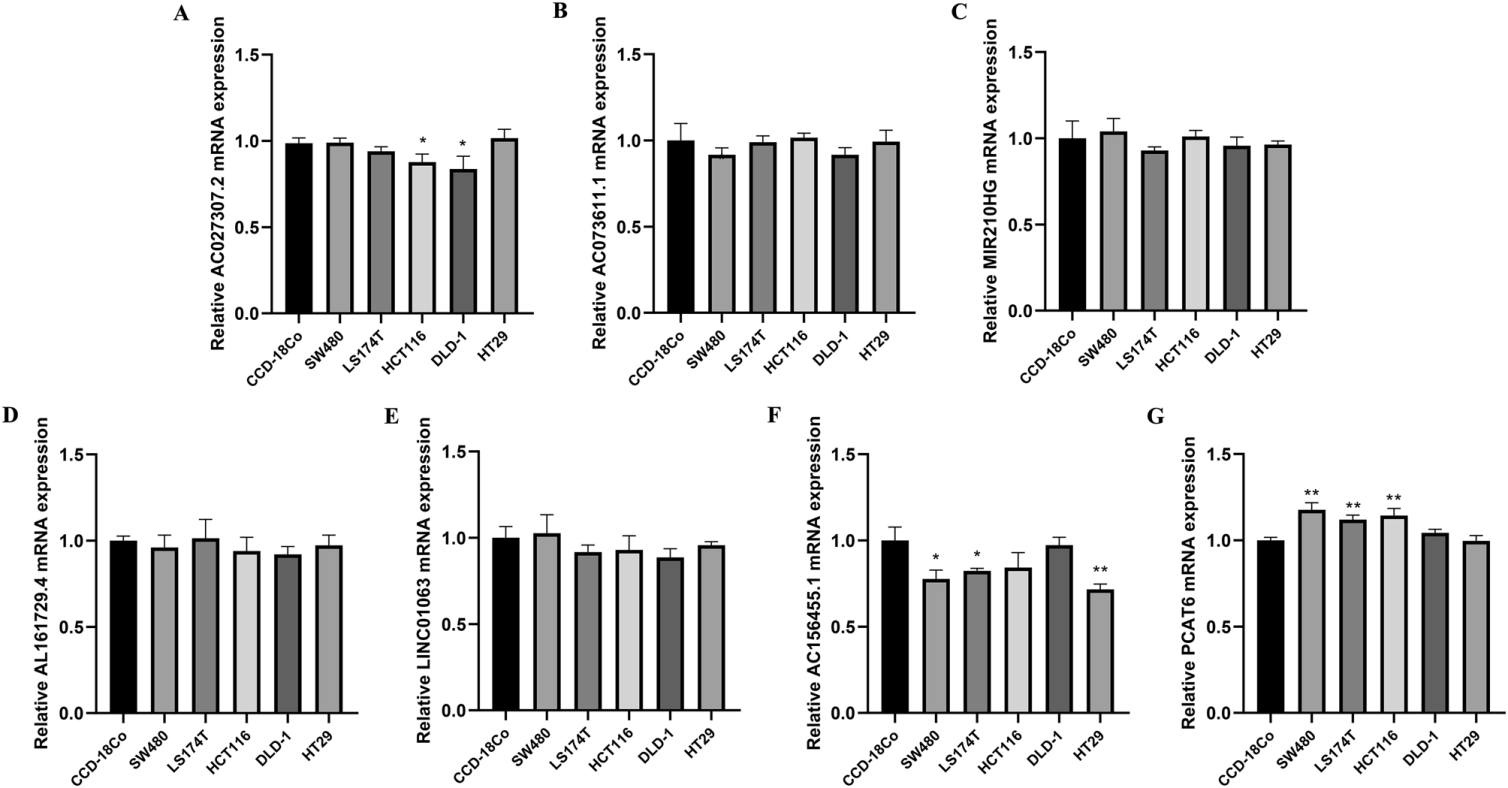
The qPCR result of seven lncRNAs in five COAD and normal colon mucosal epithelial cell lines. (**A**) qPCR result of AC027307.2; (**B**) qPCR result of AC073611.1; (**C**) qPCR result of MIR210HG; (**D**) qPCR result of AL161729.4; (**E**) qPCR result of LINC01063; (**F**) qPCR result of AC156455.1; (**G**) qPCR result of PCAT6.

We evaluated the mRNA level of seven lncRNAs (AC027307.2, AC073611.1, AC156455.1, AL161729.4, LINC01063, MIR210HG and PCAT6) in five COAD cell lines and normal colon mucosal epithelial cell line (Fig. 8). The result revealed that AC027307.2 has a lower expression level in HCT116 and DLD-1 cell lines than CCD-18Co (Fig. 8A). The mRNA level of AC073611.1, MIR210HG, AL161729.4 and LINC01063 has no statistically significant difference between cancer and normal cell lines (Fig. 8B–E). AC156455.1 mRNA level is significantly down-regulated in SW480, LS174T and HT29 cell lines (Fig. 8F). A higher mRNA level of PCAT6 is observed in SW480, LS174T and HCT116 cell lines (Fig. 8G).

## Discussion

COAD is one of the most common cancers worldwide and responsible for more than 600,000 deaths each year^17^. Many factors, such as high-fat and low-fiber diets and genetics, are now widely recognized risk factors for COAD^18–20^. Despite the progress in the development of effective diagnostic and therapeutic strategies for COAD, the lack of detection in early status and subsequent invasiveness have rendered the disease a persistent problem for susceptible populations^21^. Therefore, useful biomarkers for the early diagnosis and prognosis prediction of COAD are crucial.

Although the role of autophagy's in cancer treatment remain unclear, substantial evidence suggests the immense potential of autophagy therapy as a novel approach for COAD treatment^22^. Meanwhile, with a wide range of functional activities, lncRNAs may play a pivotal role in physiological processes, such as RNA decay, genetic regulation of gene expression, RNA splicing, and protein folding^23,24^. They can regulate many proteins that are essential to autophagy. In this study, basing on the open-access data obtained from the TCGA database (TCGA-COAD), we systematically studied the association between autophagy-related lncRNA and COAD through bioinformatics analysis. We aimed to screen signatures that are useful in predicting the development of COAD and guiding therapy strategy becaue these signature might be novel prognostic markers. To the best of our knowledge, this is the first study that focuses on the role of autophagy-related lncRNAs in COAD treatment and tumor microenvironment regulation.

First, we identified seven prognostic-related lncRNAs by respectively conducting univariate cox analysis, LASSO regression, and multivariate cox analysis. We divided the patients with COAD into high- and low-risk groups according to their median risk scores. The low-risk group had a longer OS. Through univariate and multivariate COX regression analysis, we were able to conclude that our prognostic model is efficient and independent of other clinical factors, such as TNM classification, clinical stage, age, and gender.

Next, we constructed a co-expression network of autophagy-related lncRNAs and mRNAs. Most of the nodes of the network were reported for the first time. Currently, autophagy is still an emerging field in preliminary basic studies, and the implication of lncRNAs in autophagy is extremely important^25^. For example, Liu et al. revealed that under energy stress, the lncRNA NBR2 can interact with AMPK and promotes AMPK kinase activity, subsequently activating autophagy in cancer cells^26^. Moreover, the lncRNAs HOTAIRM1, PTENP1, and MALAT1 were found to be involved in the activation of autophagy and regulation of several physiological processes and malignant phenotype of cancer cells^27–30^. By contrast, the down-regulated lncRNA Risa can improve insulin sensitivity by enhancing autophagy^31^. In COAD, on the one hand, autophagy is involved in tumor development and drug resistance^32^. On the other hand, as non-canonical regulators, lncRNAs play a pivotal role in the physiological balance of organisms by binding to a variety of molecules, such as DNA, RNA, and proteins^33^. LncRNAs can affect the physiological processes of tumor cells by interacting with autophagy-related genes and proteins. However, studies focusing on the role of lncRNAs regulating autophagy in colorectal cancer (CRC) are rare. Shan et al. revealed that the knocking down of lincRNA POU3F3 in CRC cell lines (LOVO and SW480) can significantly inhibit cell proliferation and induce G1 cell cycle arrest by activating autophagy^34^. The lncRNA HAGLROS is highly expressed in CRC and associated with decrease in OS in tumor patients^35^. The decreased expression of this lncRNA can promote apoptosis and suppress autophagy through the axis of the miR-100/ATG5 and PI3K/ AKT/mTOR pathways. The network we constructed comprehensively described the role of autophagy-related lncRNAs in COAD and can provide direction for future research. Of these seven lncRNAs, PCAT6 was reported could inhibit apoptosis by regulating anti-apoptotic proteins ARC and EZH2^36^. Besides, Perkwoska et al. revealed that the lncRNA MIR210HG might be tightly associated with autophagy, epithelial-mesenchymal transition, and cell proliferation in cell culture models of Glioblastoma^37^.

Our results showed that patients in the M1 or N1-2 stage a likely to have high risk scores. This condition partially explains the observed poor prognosis impact. Furthermore, we performed GSEA analysis to explore the difference in biological function and pathways between the high- and low-risk groups. Notably, in the high-risk phenotype, the VEGF and Notch signaling pathways were enriched. Many researchers have observed that tumor development pro-angiogenic factors, such as VEGF, basic and acidic fibroblast growth factor, tumor necrosis factor-α, and interleukin-1 are enhanced in multiple pathological processes. The persistent growth of tumor-directed capillary networks creates a favorable microenvironment, promoting cancer growth, progression, and metastasis^38^. Notch signaling is a significant regulator of sprouting angiogenesis, which is controlled by a tightly regulated balance between endothelial tip and stalk cells^39^. Moreover, the Notch site activated by adjacent vascular cells in cancer cells increases migration across endothelial cells, thus increasing metastasis in CRC patients^40^. Additionally, the expression of Jag1 in ECs can activate the Notch signal of progenitor cells and induce pericyte differentiation or further modulate the properties of cancer stem cells^41^.

Finally, we evaluated the effect of risk scores on the tumor microenvironment (stromal score and immune score), which have essential roles in COAD development^42^. Differences among the components of immune and stromal cells can substantially affect patients’ survival^42–44^. In our study, a negative relation was found between risk and tumor environment scores (stromal and immune). Meanwhile, the patients in the TGCA cohort with low immune and stromal scores may have poor prognoses. These results provide a new perspective for the poor prognosis of high-risk COAD patients.

Some limitation still exists in our study. First, the data series obtained for analysis are primarily from Western countries, and thus the results of the study may not fully apply to patients in Asian countries, given the difference in genetics between races. Second, the amount of data published in the public database is limited, and thus the clinical pathology parameters used for analysis in this study are not comprehensive and may lead to potential errors or biases.

## Conclusions

Through serial bioinformatics analysis, we identified autophagy-related lncRNAs that markedly affect the prognoses of patients. Basing on these lncRNAs, we established an effective prognosis model for predicting the OS of patients with COAD. Moreover, the co-expression network-linked autophagy-related lncRNAs and genes can provide direction for future research.

## Supplementary Information

Supplementary Figure.

Supplementary Figure.

Supplementary Information 3.

Supplementary Legends.

## Data Availability

The datasets used and/or analyzed during the current study are available from the corresponding author on reasonable request. The expression profile of 1285 autophagy-related lncRNAs has been uploaded on the website https://figshare.com/articles/dataset/lncRNAs/13490610 (figshare).

## References

[1] Meyerhardt JA, Mayer RJ (2005). Systemic therapy for colorectal cancer. N. Engl. J. Med..

[2] Haggar FA, Boushey RP (2009). Colorectal cancer epidemiology: incidence, mortality, survival, and risk factors. Clin. Colon. Rectal. Surg..

[3] Pietrzyk L, Torres A, Maciejewski R, Torres K (2015). Obesity and obese-related chronic low-grade inflammation in promotion of colorectal cancer development. Asian Pacific J. Cancer Prev.: APJCP.

[4] Elinav E (2013). Inflammation-induced cancer: crosstalk between tumours, immune cells and microorganisms. Nat. Rev. Cancer.

[5] Levine B, Kroemer G (2008). Autophagy in the pathogenesis of disease. Cell.

[6] White E (2015). The role for autophagy in cancer. J. Clin. Investig..

[7] Xiao T (2018). RACK1 promotes tumorigenicity of colon cancer by inducing cell autophagy. Cell Death Dis..

[8] Rigoutsos I (2017). N-BLR, a primate-specific non-coding transcript leads to colorectal cancer invasion and migration. Genome Biol..

[9] Fang C (2017). Long non-coding RNA HNF1A-AS1 mediated repression of miR-34a/SIRT1/p53 feedback loop promotes the metastatic progression of colon cancer by functioning as a competing endogenous RNA. Cancer Lett..

[10] Wang L (2018). Long noncoding RNA B3GALT5-AS1 suppresses colon cancer liver metastasis via repressing microRNA-203. Aging.

[11] Chi, Y., Wang, D., Wang, J., Yu, W. & Yang, J. Long Non-Coding RNA in the Pathogenesis of Cancers. *Cells* **8**, doi:10.3390/cells8091015 (2019). 10.3390/cells8091015 PMC6770362 31480503

[12] Kulasingam V, Diamandis EP (2008). Strategies for discovering novel cancer biomarkers through utilization of emerging technologies. Nat. Clin. Pract. Oncol..

[13] Zerbino DR (2018). Ensembl 2018. Nucleic Acids Res..

[14] Shannon P (2003). Cytoscape: a software environment for integrated models of biomolecular interaction networks. Genome Res..

[15] Kanehisa M, Furumichi M, Sato Y, Ishiguro-Watanabe M, Tanabe M (2021). KEGG: integrating viruses and cellular organisms. Nucleic Acids Res..

[16] Subramanian A (2005). Gene set enrichment analysis: a knowledge-based approach for interpreting genome-wide expression profiles. Proc. Natl. Acad. Sci. USA.

[17] Ferlay J (2015). Cancer incidence and mortality worldwide: sources, methods and major patterns in GLOBOCAN 2012. Int. J. Cancer.

[18] Gerner EW, Bruckheimer E, Cohen A (2018). Cancer pharmacoprevention: Targeting polyamine metabolism to manage risk factors for colon cancer. J. Biol. Chem..

[19] O'Keefe SJ (2016). Diet, microorganisms and their metabolites, and colon cancer. Nat. Rev. Gastroenterol. Hepatol..

[20] Mody K, Bekaii-Saab T (2018). Clinical Trials and Progress in Metastatic Colon Cancer. Surg. Oncol. Clin. N. Am..

[21] Stein A, Hiemer S, Schmoll HJ (2011). Adjuvant therapy for early colon cancer: current status. Drugs.

[22] Devenport, S. N. & Shah, Y. M. Functions and Implications of Autophagy in Colon Cancer. *Cells* **8**, doi:10.3390/cells8111349 (2019). 10.3390/cells8111349 PMC6912527 31671556

[23] Mercer TR, Dinger ME, Mattick JS (2009). Long non-coding RNAs: insights into functions. Nat. Rev. Genet..

[24] Chen YG, Satpathy AT, Chang HY (2017). Gene regulation in the immune system by long noncoding RNAs. Nat. Immunol..

[25] Yang L, Wang H, Shen Q, Feng L, Jin H (2017). Long non-coding RNAs involved in autophagy regulation. Cell Death Dis..

[26] Liu X (2016). LncRNA NBR2 engages a metabolic checkpoint by regulating AMPK under energy stress. Nat. Cell Biol..

[27] Zhuo C, Jiang R, Lin X, Shao M (2017). LncRNA H19 inhibits autophagy by epigenetically silencing of DIRAS3 in diabetic cardiomyopathy. Oncotarget.

[28] Tang S (2016). An artificial lncRNA targeting multiple miRNAs overcomes sorafenib resistance in hepatocellular carcinoma cells. Oncotarget.

[29] Pawar K, Hanisch C, Palma Vera SE, Einspanier R, Sharbati S (2016). Down regulated lncRNA MEG3 eliminates mycobacteria in macrophages via autophagy. Sci. Rep..

[30] Chen CL (2015). Suppression of hepatocellular carcinoma by baculovirus-mediated expression of long non-coding RNA PTENP1 and MicroRNA regulation. Biomaterials.

[31] Wang Y (2016). Down-regulation of Risa improves insulin sensitivity by enhancing autophagy. FASEB J..

[32] Santana-Codina N, Mancias JD, Kimmelman AC (2017). The Role of Autophagy in Cancer. Annu. Rev. Cancer Biol..

[33] Ferrè F, Colantoni A, Helmer-Citterich M (2016). Revealing protein-lncRNA interaction. Brief. Bioinform..

[34] Shan TD (2016). Knockdown of linc-POU3F3 suppresses the proliferation, apoptosis, and migration resistance of colorectal cancer. Oncotarget.

[35] Zheng Y, Tan K, Huang H (2019). Long noncoding RNA HAGLROS regulates apoptosis and autophagy in colorectal cancer cells via sponging miR-100 to target ATG5 expression. J. Cell. Biochem..

[36] Huang W (2019). Long noncoding RNA PCAT6 inhibits colon cancer cell apoptosis by regulating anti-apoptotic protein ARC expression via EZH2. Cell Cycle.

[37] Witusik-Perkowska M, Jaskólski DJ, Liberski PP, Szemraj J (2020). If artificial in vitro microenvironment can influence tumor drug resistance network via modulation of lncRNA expression?-Comparative analysis of glioblastoma-derived cell culture models and initial tumors in vivo. Cell. Mol. Neurobiol..

[38] Siveen KS (2017). Vascular endothelial growth factor (VEGF) signaling in tumour vascularization: potential and challenges. Curr. Vasc. Pharmacol..

[39] Hellström M (2007). Dll4 signalling through Notch1 regulates formation of tip cells during angiogenesis. Nature.

[40] Sonoshita M (2011). Suppression of colon cancer metastasis by Aes through inhibition of Notch signaling. Cancer Cell.

[41] Patenaude A (2015). A novel population of local pericyte precursor cells in tumor stroma that require Notch signaling for differentiation. Microvasc. Res..

[42] Pagès F (2018). International validation of the consensus Immunoscore for the classification of colon cancer: a prognostic and accuracy study. Lancet (London, England).

[43] Zhou R (2019). Immune cell infiltration as a biomarker for the diagnosis and prognosis of stage I-III colon cancer. Cancer Immunology, Immunotherapy : CII.

[44] Hamada, T. *et al.* Vitamin D status after colorectal cancer diagnosis and patient survival according to immune response to tumour. *Eur. J. Cancer (Oxford, England : 1990)* **103**, 98–107, doi:10.1016/j.ejca.2018.07.130 (2018). 10.1016/j.ejca.2018.07.130 PMC6195453 30219720

