# Supplementary figures and images for: Identification of novel autophagy-related lncRNAs associated with a poor prognosis of colon adenocarcinoma through bioinformatics analysis
Source: Sci Rep. 2021 Apr 13;11:8069. doi: 10.1038/s41598-021-87540-0 (PMC8044244; doi:10.1038/s41598-021-87540-0)

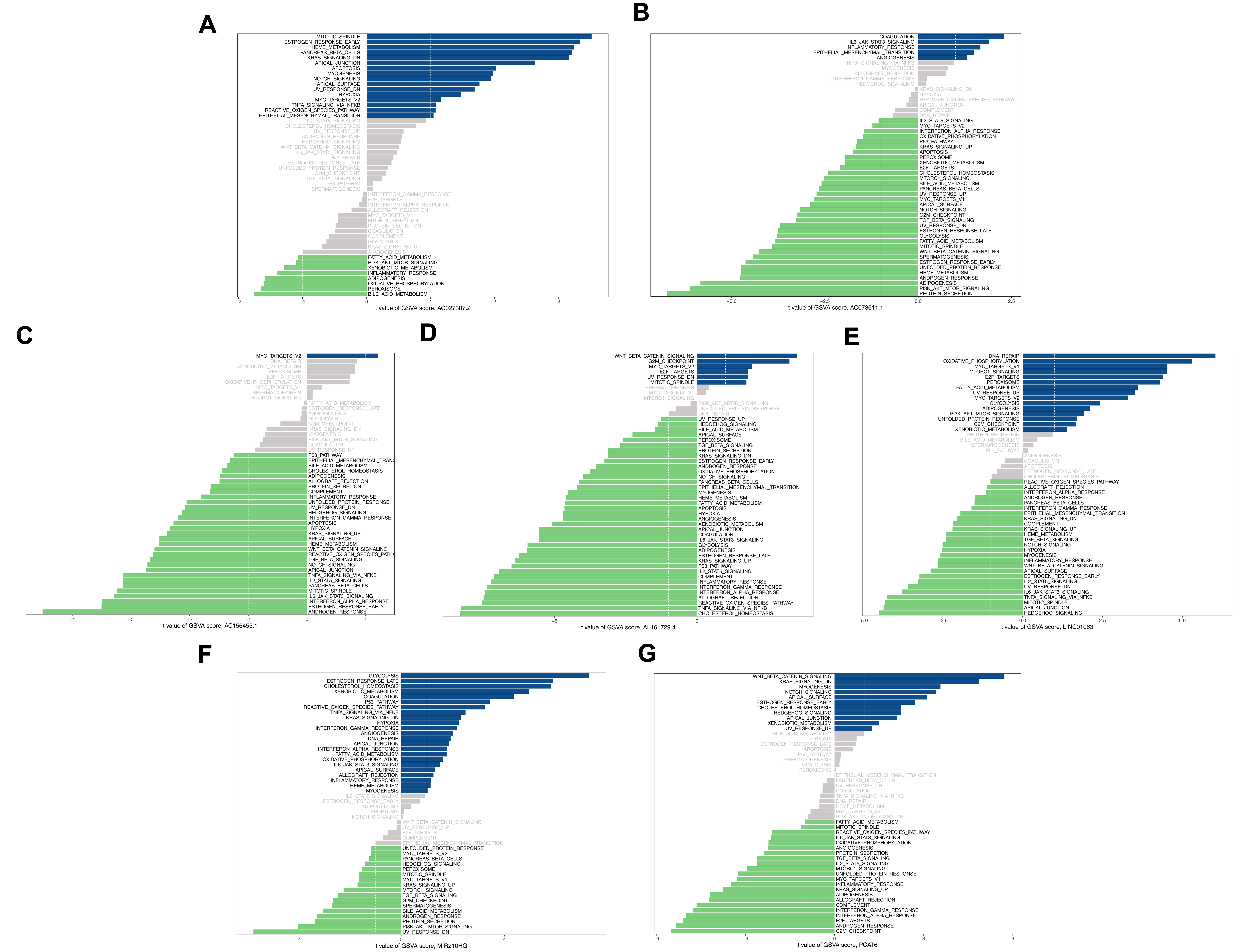

Supplement: Supplementary file 1 — Supplementary Figure. [file 41598_2021_87540_MOESM1_ESM.tif]

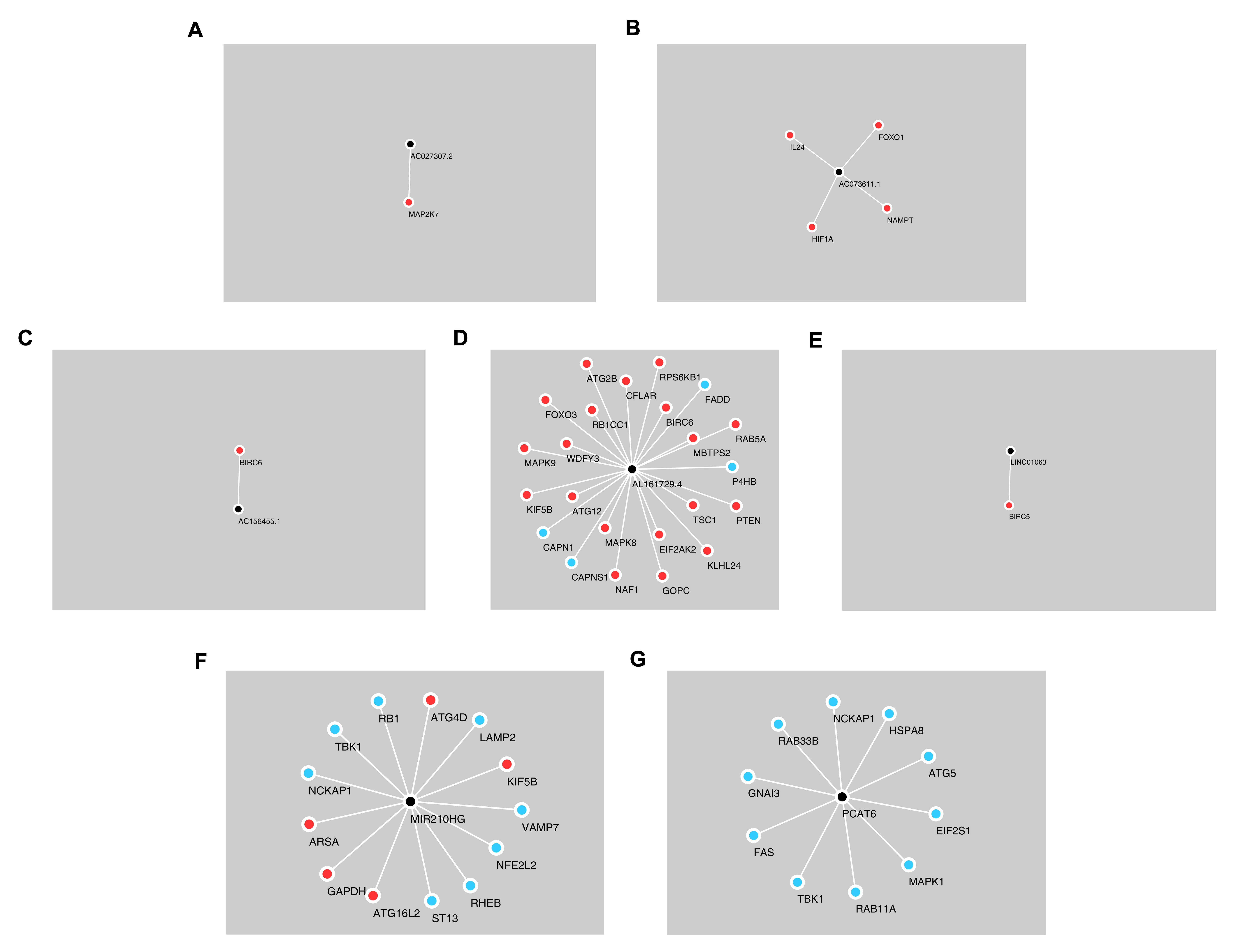

Supplement: Supplementary file 2 — Supplementary Figure. [file 41598_2021_87540_MOESM2_ESM.tif]
