# Supplementary material for: Identification of novel autophagy-related lncRNAs associated with a poor prognosis of colon adenocarcinoma through bioinformatics analysis
Source: Sci Rep. 2021 Apr 13;11:8069. doi: 10.1038/s41598-021-87540-0 (PMC8044244; doi:10.1038/s41598-021-87540-0)
Supplement: Supplementary file 3 — Supplementary Information 3. [file 41598_2021_87540_MOESM3_ESM.docx]

**Identification of novel autophagy-related lncRNAs associated with a poor prognosis of colon adenocarcinoma through bioinformatics analysis**

**Dejun Wu^1*^, Zhenhua Yin^2*^, Yisheng Ji^4*^, Lin Li^4*^, Yunxin Li^4^, Fanqiang Meng^5*^,, Xiaohan Ren^3#^, Ming Xu^1#^**

| **Table S1. the 231 prognosis-related genes** |
| --- |
| AMBRA1, APOL1, ARNT, ARSA, ARSB, ATF4, ATF6, ATG10, ATG12, ATG16L1, ATG16L2, ATG2A, ATG2B, ATG3, ATG4A, ATG4B, ATG4C, ATG4D, ATG5, ATG7, ATG9A, ATG9B, ATIC, BAG1, BAG3, BAK1, BAX, BCL2, BCL2L1, BECN1, BID, BIRC5, BIRC6, BNIP1, BNIP3, BNIP3L,, C12orf44, C17orf88, CALCOCO2, CAMKK2, CANX, CAPN1, CAPN10, CAPN2, CAPNS1, CASP1, CASP3, CASP4, CASP8, CCL2, CCR2, CD46, CDKN1A, CDKN1B, CDKN2A, CFLAR, CHMP2B, CHMP4B, CLN3, CTSB, CTSD, CTSL1, CX3CL1, CXCR4, DAPK1, DAPK2, DDIT3, DIRAS3  DLC1, DNAJB1, DNAJB9, DRAM1, EDEM1, EEF2, EEF2K, EGFR, EIF2AK2, EIF2AK3, EIF2S1, EIF4EBP1, EIF4G1, ERBB2, ERN1, ERO1L  FADD, FAM48A, FAS, FKBP1A, FKBP1B, FOS, FOXO1, FOXO3, GAA  GABARAP, GABARAPL1, GABARAPL2, GAPDH, GNAI3, GNB2L1  GOPC, GRID1, GRID2, HDAC1, HDAC6, HGS, HIF1A, HSP90AB1, HSPA5, HSPA8, HSPB8, IFNG, IKBKB, IKBKE, IL24, IRGM, ITGA3, ITGA6, ITGB1, ITGB4, ITPR1, KIAA0226, KIAA0652, KIAA0831, KIF5B  KLHL24, LAMP1, LAMP2, MAP1LC3A, MAP1LC3B, MAP1LC3C, MAP2K7, MAPK1, MAPK3, MAPK8, MAPK8IP1, MAPK9, MBTPS2, MLST8, MTMR14, MTOR, MYC, NAF1, NAMPT, NBR1, NCKAP1, NFE2L2, NFKB1, NKX2-3, NLRC4, NPC1, NRG1, NRG2, NRG3, P4HB, PARK2, PARP1, PEA15, PELP1, PEX14, PEX3, PIK3C3, PIK3R4, PINK1  PPP1R15A, PRKAB1, PRKAR1A, PRKCD, PRKCQ, PTEN, PTK6, RAB11A, RAB1A, RAB24, RAB33B, RAB5A, RAB7A, RAC1, RAF1, RB1  RB1CC1, RELA, RGS19, RHEB, RPS6KB1, RPTOR, SAR1A, SERPINA1  SESN2, SH3GLB1, SIRT1, SIRT2, SPHK1, SPNS1, SQSTM1, ST13, STK11, Symbol, TBK1, TM9SF1, TMEM49, TMEM74, TNFSF10, TP53, TP53INP2, TP63, TP73, TSC1, TSC2, TUSC1, ULK1, ULK2, ULK3, USP10, UVRAG, VAMP3, VAMP7, VEGFA, WDFY3, WDR45, WDR45L, WIPI1, WIPI2, ZFYVE1 |
