# Supplementary material for: Identification of novel autophagy-related lncRNAs associated with a poor prognosis of colon adenocarcinoma through bioinformatics analysis
Source: Sci Rep. 2021 Apr 13;11:8069. doi: 10.1038/s41598-021-87540-0 (PMC8044244; doi:10.1038/s41598-021-87540-0)
Supplement: Supplementary file 4 — Supplementary Legends. [file 41598_2021_87540_MOESM4_ESM.docx]

**Figure S1.** GSVA analysis of seven lncRNAs for potential signaling pathway.

**Notes:** The dark blue terms are up-regualted pathway, and green terms are down-regulated pathway; **A:** GSVA analysis of AC027307.2; **B:** GSVA analysis of AC073611.1; **C:** GSVA analysis of AC156455.1; **D:** GSVA analysis of AL161729.4; **E:** GSVA analysis of LINC01063; **F:** GSVA analysis of MIR210HG; **G:** GSVA analysis of PCAT6.

**Figure S2.** The protein-coding genes interacted with seven lncRNAs.

**Notes:** The red nodes are positively correlated with lncRNAs, and blue nodes are negatively correlated with lncRNAs; **A:** The interaction between protein-coding genes and AC027307.2; **B:** The interaction between protein-coding genes and AC073611.1; **C:** The interaction between protein-coding genes and AC156455.1; **D:** The interaction between protein-coding genes and AL161729.4; **E:** The interaction between protein-coding genes and LINC01063; **F:** The interaction between protein-coding genes and MIR210HG; **G:** The interaction between protein-coding genes and PCAT6.
